# Red algae industrial residues as a sustainable carbon platform for the co-production of poly-3-hydroxybutyrate and gluconic acid by *Halomonas boliviensis*

**DOI:** 10.3389/fbioe.2022.934432

**Published:** 2022-10-10

**Authors:** Maryna Bondar, Filipa Pedro, M. Conceição Oliveira, M. Manuela R. da Fonseca, M. Teresa Cesário

**Affiliations:** ^1^ iBB- Institute for Bioengineering and Biosciences, Bioengineering Department, Instituto Superior Técnico, Universidade de Lisboa, Lisboa, Portugal; ^2^ Associate Laboratory i4HB, Institute for Health and Bioeconomy, Instituto Superior Técnico, Universidade de Lisboa, Lisboa, Portugal; ^3^ Centro de Química Estrutural, Institute of Molecular Sciences, Instituto Superior Técnico, Universidade de Lisboa, Lisboa, Portugal

**Keywords:** *Gelidium corneum*, *Gelidium sesquipedale*, gluconic acid, *Halomonas boliviensis*, industrial seaweed residues, seaweed hydrolysates

## Abstract

Polyhydroxyalkanoate (PHA) production using halophilic bacteria has been revisited because less severe operational conditions with respect to sterility can be applied, also alleviating production costs. *Halomonas boliviensis* was selected because it is a moderate halophile able to grow and attain high poly-3-hydroxybutyrate (P3HB) contents under 5–45 g/L NaCl concentrations, conditions that discourage microbial contamination. Industrial residues of the red alga *Gelidium corneum* after agar extraction were used as sugar platform to reduce costs associated with the carbon source. These residues still comprise a high carbohydrate content (30–40% w/w) of mainly cellulose, and their hydrolysates can be used as substrates for the bioproduction of value-added products. Preliminary assays using glucose were carried out to determine the best conditions for growth and P3HB production by *H. boliviensis* in bioreactor fed-batch cultivations. Two strategies were addressed, namely nitrogen or phosphorus limitation, to promote polymer accumulation. Similar P3HB cell contents of 50% (g_polymer_/g_CDW_) and yields *Y*_*P3HB/glucose*_ of 0.11–0.15 g _polymer_/g _glucose_ were attained under both conditions. However, higher specific productivities were reached under P-limitation, and thus, this strategy was adopted in the subsequent study. Two organic acids, resulting from glucose metabolism, were identified to be gluconic and 2-oxoglutaric acid. Reducing the oxygen concentration in the cultivation medium to 5% sat was found to minimize organic acid production and enhance the yield of polymer on sugar to 0.20 g_P3HB_/g_glucose_. Finally, fed-batch cultivations using *G. corneum* hydrolysates as the only C-source achieved an overall volumetric productivity of 0.47 g/(L.h), 40% polymer accumulation, and negligible gluconic acid production.

## 1 Introduction

Biological processes for the production of polyhydroxyalkanoates need to focus on alternative, residual carbon sources to decrease production costs and turn these microbial polyesters more competitive in the plastic market. In addition, the operation of continuous cultivations in open systems should be considered to save sterilization costs and allow the use of cheaper materials for the construction of bioreactors and ancillary equipment. Aiming at this, halophilic bacteria have been revisited due to their importance to industrial biotechnology. Their tolerance to high NaCl concentrations and high pH values in the cultivation media facilitates the operation of continuous and open fermentations without the problem of contamination (Yin et al., 2015). Among halophiles, *Halomonas* spp. are moderate halophilic bacteria that grow under salt concentrations ranging from 3 to 15% (w/v) and can tolerate 0–25% (w/v) salt (Yin et al., 2015). To thrive in saline conditions, most *Halomonas* accumulate low molecular weight, water-soluble compounds, called “compatible solutes” or osmolytes to maintain cellular osmotic balance. These organic compounds may belong to many different families, for example, polyols, sugars, amino acids, betaines, ectoines, and their derivatives, among others (Guzmán et al., 2009).

In addition to osmolytes, *Halomonas* spp. has the ability to accumulate PHAs as a reservoir of energy and reducing equivalents when the carbon source is in excess and another nutrient is limited (Steinbüchel and Füchtenbusch, 1998). Several bacterial species of the genus *Halomonas* such as *H. boliviensis* (Quillaguamán et al., 2004), *H. elongata* (Mothes et al., 2008), *H. bluephagenesis* TD1 (Tan et al., 2011; Shen et al., 2018), *H. halophila* (Kucera et al., 2018), and *H. campaniensis LS21* (Yue et al., 2014) have been reported to accumulate PHAs.

*Halomonas boliviensis* was selected in this study due to its high polymer accumulation capacity (50%–88% w/w) (Quillaguamán et al., 2008) and ability to metabolize different carbohydrates and short-chain fatty acids namely maltose and oligosaccharides of starch (Quillaguamán et al., 2005), glucose, sucrose, butyric acid, sodium acetate (Quillaguamán et al., 2006), and galactose (Tuma et al., 2020). Cultivations in bench-scale bioreactors run in the fed-batch mode have been performed with *H. boliviensis* using nitrogen (Quillaguamán et al., 2008; García-Torreiro et al., 2016) or oxygen (García-Torreiro et al., 2016) limitation strategies to promote polymer production. Maximum polymer productivities of 1.2 g/(L.h)were attained under N and low oxygen supply by García-Torreiro et al. (2016), while Quillaguaman and coauthors attained productivities of 1.1 g/(L.h) under conditions of nitrogen limitation.

Organic acids are considered to be intermediates or end products of cellular metabolism; however, only for a few acids, the microbiological production process presents itself as an economic alternative to chemical synthesis. Gluconic acid is commonly used in the food industry; it is soluble in water, nontoxic, and presents good chelation capacity at high pH (Goldberg and Rokem, 2009). Current commercial production occurs with *Aspergillus niger* under submerged state fermentation (Yadav and Chauhan, 2022). However, the production of this acid was already reported in several bacterial strains such as *Pseudomonas* and *Acetobacter*. By bacteria, the production of gluconic acid is induced by high glucose concentrations (Ramachandran et al., 2006). To date, no studies have reported the ability of *Halomonas* spp. to produce gluconic acid.

Residues of the red seaweed *Gelidium corneum* (previously called *Gelidium sesquipedale*) from an agar extraction plant with a carbohydrate content of approximately 30–40% w/w (Tuma et al., 2020) are a potential carbon platform yet to be exploited. These residues contain mainly cellulose and agar leftovers, and upon hydrolysis, they release simple sugars that can be used for growth and P3HB production by *H. boliviensis*. In a previous study, P3HB cell contents of 41% (w/w) and a yield (*Y*_*P/S*_) of 0.16 g _polymer_/g_glucose_ were attained in shake flask cultivations with glucose-rich *G. corneum* hydrolysates, thus validating the proof-of-concept (Tuma et al., 2020).

In this work, hydrolysates from *Gelidium* residues were utilized as C-source for P3HB production in bioreactor cultivations operating in the fed-batch mode to achieve high productivities. A preliminary study was carried out with glucose to determine the best strategy to attain high P3HB productivities and yields with *H. boliviensis.* Coproduction of P3HB and gluconic acid was observed in cultivations both under nitrogen- and phosphorous-limiting conditions. This is the first time that the ability of *Halomonas boliviensis* to synthesize gluconic acid is reported.

## 2 Materials and methods

### 2.1 Chemicals and feedstock

Commercial glucose (dextrose monohydrate, Dextropam 100 kindly supplied by Copam-Portugal) was used as the substrate in bioreactor cultivation; D (+) glucose anhydrous 99.5% (Fisher Chemicals), gluconic acid sodium salt 99% (Sigma), and 2-oxoglutaric acid for biochemistry (Merck) were used as standards in the analyses by HPLC. For the identification of metabolites by LC-MS, the aqueous solutions and the mobile phases were prepared with water, acetonitrile, and formic acid, grade LC-MS Optima (Fisher Scientific)*.*

The *Gelidium corneum* residues were supplied by Iberagar–Sociedade Luso-Espanhola de Colóides Marinhos S.A., and its composition (expressed in g/100 g of raw material in oven-dry basis ±standard deviation based on three replicate determinations) was 35.26 ± 0.87 of ash, 16.00 ± 0.94 of crude protein, 19.24 ± 0.88 of glucan, and 5.24 ± 0.06 of galactan.

The hydrolysates from the *Gelidium* residues were prepared and delivered by the Centre of Biological Engineering (CBE), University of Minho, Portugal (project partners in PTDC/BII-BIO/29242/2017).

### 2.2 Microorganisms and strain storage

*Halomonas boliviensis* DSM 15516, a halophilic bacterium able to produce P3HB from glucose and galactose, the sugars present in *Gelidium corneum* hydrolysates, was used in this work.

Cultures of *H. boliviensis* were stored at −80°C in 2-ml sterile cryotubes containing 300 µl of sterilized pure glycerol and 1,500 µl of a previously grown liquid culture in the late exponential phase. The latter was prepared by growing the cells in a seed medium supplemented with 20 g/L of glucose at 30°C in an orbital incubator (Aralab, AGITORB 200-Portugal) at 170 rpm for 24 h.

### 2.3 Seed medium and inoculum preparation

The inoculum for the bioreactor assays was prepared with the following medium composition (the seed medium): 20 g/L of glucose, 45 g/L NaCl, 25 ml/L of a 100 g/L MgSO_4_.7H_2_O solution, 0.55 g/L K_2_HPO_4_, 2.3 g/L NH_4_Cl, 15 g/L Tris, 3 g/L monosodium glutamate (MSG), and 0.005 g/L FeSO_4_.7H_2_O. All the medium components except NaCl, MgSO_4_.7H_2_O and glucose were dissolved in distilled water, and the pH was adjusted to 7.5 with HCl (5M). This mixture was then sterilized in the autoclave at 121°C for 20 min. Concentrated solutions of NaCl (300 g/L), MgSO_4_.7 H_2_O (100 g/L) and glucose (500 g/L) were prepared and sterilized separately to avoid the formation of precipitates during the sterilization process. These solutions were then added to the medium to meet the desired concentrations.

The contents of two cryovials were added to 500-ml shake flasks containing the seed medium to attain a final working volume of 65 ml. Incubation occurred at 30°C in an orbital shaker at 170 rpm and lasted 18 h. At this point, the culture was in the late exponential growth phase, and the O.D._600 nm_ reached a value of approximately 5.

Two inoculum flasks were prepared to reach a total volume of 130 ml, which corresponds to 10% (v/v) of the initial working volume of the bioreactor.

### 2.4 Bioreactor mineral medium

The initial composition of the mineral medium in the bioreactor assays is given in Table 1. Depending on the strategy to promote the P3HB production, different initial K_2_HPO_4_ and NH_4_Cl concentrations were used. Except for MgSO_4_.7H_2_O, NaCl and glucose, the remaining medium components were sterilized in the bioreactor. Before sterilization, the pH was adjusted to 7.5 with a 2.5 M KOH (PanReac) solution. Concentrated solutions of MgSO_4_.7H_2_O (100 g/L), NaCl (300 g/L) and glucose (500 g/L) were autoclaved separately and added to the medium aseptically to attain the desired concentrations.

**TABLE 1:** Composition of the initial medium and feed in fed-batch cultivations.

	Initial medium composition	
Component	N-lim condition (g/L)	P-lim condition (g/L)	Feed (g/L)
NaCl	45.0	45.0	45
MgSO_4_.7H_2_O	5.0	5.0	5.0
K_2_HPO_4_	4.5	1.5	-
NH_4_Cl	3.0	7.0	-
MSG	20.0	20.0	-
FeSO_4_.7H_2_O	0.005	0.005	0.125
Glucose	25.0	25.0	600

### 2.5 Hydrolysates from industrial *Gelidium* residues

The optimized protocol to produce the liquor from *Gelidium* residues included a hydrothermal pretreatment at 170°C for 20 min, at a solid loading of 20% (w/v) in distilled water (Gomes-Dias et al., 2020). After this step, the soluble galactan fraction and the hydroxymethyl furfural (HMF) that might have been produced in the autohydrolysis step were discarded upon filtration of the whole slurry. The pretreated solid was subjected to enzymatic hydrolysis using a Cellic cTec2 commercial cocktail, at 15 FPU/g, pH 5, 50°C and 72 h to obtain fermentable glucose. The liquid fraction was separated and concentrated to approx. 358 g/L glucose using a vacuum stove at 60°C. A concentration of 0.28 g/L HMF in the final concentrated hydrolysate was detected.

### 2.6 Bioreactor cultivations

Fed-batch cultivations were carried out in 2-L STRs (New Brunswick BioFlo 115) operated by BioCommand Batch Control software which enabled control, monitoring and data acquisition. The pH of the cultivation medium was controlled at 7.5 with 2.5 M NaOH (when using N-limiting conditions) or 30% (v/v) NH_4_OH (in P-limiting assays) and 2 M of H_2_SO_4_, and the temperature was set to 30°C. The dissolved oxygen (DO) set-point used was 5, 20, or 45% of the saturation concentration. To maintain the DO at the established value, the aeration rate used was 1.0 L/min or 2.6 L/min, and the agitation speed was set in cascade with the DO. The maximum agitation speed allowed by the equipment is 1,200 rpm. The initial volume of the fed-batch cultivations was 1.3 L, including all medium components and the inoculum. The initial medium composition is given in Table 1. A 600 g/L solution of glucose (V_feed_ = 500 ml) supplemented with NaCl, magnesium and iron was used as feed (Table 1). In the assays using P-limitation, to avoid an initially high ammonia concentration (7 g/L; Table 1), it was decided to add different pulses of a concentrated NH_4_Cl solution in the first 20 h of cultivation. This strategy has been suggested by Quillaguamán et al. (2008). The feed of glucose (Table 1) was then added in pulses to the bioreactor. At approximately 10 h cultivation, a pulse of 10 ml feed was programmed to avoid glucose exhaustion during the first night. Thereafter and for the next 12 h, 10 ml feed pulses were added every hour.

When algal hydrolysates were evaluated as the substrate, a volume of hydrolysate was used to attain an initial glucose concentration of 25 g/L in the batch phase, and K_2_HPO_4_ was supplemented to the hydrolysate to work in P-limiting conditions (Table 1). This mixture was autoclaved in the bioreactor. After cooling, concentrated solutions of MSG, NaCl, MgSO_4_.7H_2_O, and FeSO_4_.7H_2_O were added according to Table 1. Again, to avoid high ammonia concentrations in the onset of cultivation, a concentrated NH_4_Cl solution was added in the first 20 h in pulses of 20 ml. Thereafter, 0.5 L of the same hydrolysate after supplementation with NaCl, MgSO_4_.7H_2_O, and FeSO_4_.7H_2_O (Table 1) was used as feed to supply glucose.

Culture samples were periodically harvested to monitor biomass, polymer, sugar, phosphate, and ammonium concentrations during the cultivation.

### 2.7 Analytical methods

#### 2.7.1 Biomass concentration

The cellular growth was monitored offline by measuring the optical density (OD) of culture samples at 600 nm in a double beam spectrophotometer (Hitachi U-2000), using 3-ml glass cuvettes with an optical path length of 1 cm. Deionized water was used as a reference.

The cell dry weight (*CDW*) was determined by centrifuging at 9,168 g for 5 min (Sigma 1-15 P microcentrifuge) 1.2 ml aliquots of culture samples collected in previously dried and weighed microtubes. The supernatant was rejected, and the pellet was washed with deionized water and then dried at 60°C in a Memmert oven (Model 400) until constant weight.

#### 2.7.2 Sugar, phosphate, and organic acid quantification

A high-performance liquid chromatography (HPLC) Hitachi LaChrom Elite system was used for offline determination of glucose, gluconic, and 2-oxoglutaric acids, as well as phosphate. It was equipped with a Rezex ROA-Organic acid H + 8% (300 mm × 7.8 mm) column, an autosampler (Hitachi LaChrom Elite L-2200), an HPLC pump (Hitachi LaChrom Elite L-2130), a Hitachi L-2490 refraction index (RI) detector for sugars and phosphate, and a Hitachi L-2420 UV-VIS detector for organic acids. A column heater (Croco-CIL 100-040-220P; 40 cm × 8 cm x 8 cm; 30–99°C) was connected externally to the HPLC system. The column was kept at 65°C. A 5 mM solution of H_2_SO_4_ was used as the mobile phase at an elution rate of 0.5 ml/min.

Calibration curves were obtained for glucose, gluconic and 2-oxoglutaric acids and phosphate. Glucose and gluconic acid showed similar retention times, which led to an incorrect determination of the glucose concentration using the RI detector whenever gluconic acid was present. To solve this problem, glucose was determined using a D-glucose assay kit (the GOPOD format) from Megazyme. The gluconic acid concentration was determined using a UV detector at a wavelength of 210 nm.

#### 2.7.3 Poly-3-hydroxybutyrate quantification

For P3HB quantification, depending on the estimated concentration of polymer produced, an adequate aliquot of culture medium was withdrawn to a microtube (1.2, 0.6, or 0.3 ml) and centrifuged. The pellet was frozen after being washed with distilled water. This pellet was then subjected to acidic methanolysis to convert the polymer into stable and volatile hydroxycarboxylic acid methyl esters, to be further detected by gas chromatography. The methanolysis protocol consisted in adding 1 ml of chloroform to the cell pellet; the mixture was resuspended and transferred to a glass tube. Then, 1 mL of a solution containing 97 ml methanol, 3 ml 96% H_2_SO_4_, and 330 µl of hexanoic acid was added to each tube. After vortexing and closing the tubes with caps containing a Teflon interior coating, the tubes were placed in an oven (Memmert model 200) at 100°C for 5 h. After cooling, the reaction was stopped by adding 1 ml of Na_2_CO_3_ solution (60 g/L). Thereafter, the tubes were vortexed and by centrifugation (5 min at 2697 g in a Heraeus Labofuge 200 system from Thermo Scientific), the organic and the aqueous phases were separated. An aliquot of 200 µl of the bottom phase (chloroform) was withdrawn and added to a GC vial. The vials were stored at −18°C until analysis. Determination of the P3HB concentration was carried out by gas chromatography (Agilent Technologies 5890 series II) using a FID detector and a 7683B injector. The capillary column was an HP-5 column from Agilent J&W Scientific, 30 m in length and 0.32 mm in internal diameter. The oven, injector, and detector were kept at constant temperatures of 60°C, 120°C, and 150°C, respectively. Data acquisition and integration were performed by a Shimadzu CBM-102 Communication Bus Module and Shimadzu GC solution software (version 2.3), respectively. Calibration curves were obtained using samples of P(3HB) produced previously (purity 98.2%), which were subjected to the same methylation process as the cells. Peak identification was achieved using standard 3-methyl hydroxybutyrate (Sigma).

#### 2.7.4 Ammonium quantification

The concentration of ammonium ions present in the fermentation broth was determined using the phenate method (APHA et al., 2005) (Standard Methods, 1999 for the Examination of Water and Wastewater Part 4000 inorganic nonmetallic constituents). The optical density (OD) of samples at 640 nm was measured in a double beam spectrophotometer (Hitachi U-2000) in 1-ml plastic cuvettes with an optical path length of 1 cm. The reference used was the mineral medium with the composition described in Table 1, except for NH_4_Cl, and it was submitted to the same treatment as the samples. For quantification, the supernatant of the samples was diluted from 1:25 to 1:2500 with deionized water to a final volume of 5 ml. A measure of 200 µl of phenol solution, 200 µl of sodium nitroprusside, and 500 µl of the oxidizing solution was then added. The samples were placed in a dark environment for 1 h at room temperature for color to develop and were analyzed within 24 h. Phenol solution, sodium nitroprusside (0.5% w/v), and the oxidizing solution were prepared according to the protocol mentioned previously.

The calibration curve was obtained for the working range of 0.1–0.6 mg/L. The solution used to construct the calibration curve was the medium (Table 1) after dilution of 1:2500.

#### 2.7.5 Organic acid identification by liquid chromatography–high-resolution mass spectrometry

Aqueous solutions of gluconic and 2-oxoglutaric acid standards and aliquots of cultivation media of *Halomonas boliviensis* in glucose from bioreactor fed-batch assays were analyzed on a UHPLC Elute interfaced using a QqTOF Impact II mass spectrometer equipped with an ESI source (Bruker Daltonics, Bremen, Germany). Chromatographic separation was carried out on a Luna Omega C18 Polar column (150 mm × 2.1 mm, 1.6 μm particle size; Phenomenex), using a gradient elution of 0.1% formic acid in water (mobile phase A) and acetonitrile (mobile phase B), at a flow rate of 270 μl/min. The elution conditions were as follows: 0–0.5 min, isocratic 0% B; 0.5–3 min, linear gradient to 10% B; 3–6 min, linear gradient to 100% B; 6–9 min isocratic 100% B; 9–10 min, linear gradient to 0% B; and 10–15 min, isocratic to 0% B. The column and the autosampler were maintained at 35°C and 8°C, respectively. The high-resolution mass spectra were acquired in both ESI-positive and -negative modes. The optimized parameters were set as follows: ion spray voltage, +4.5/−2.5 kV; end plate offset, 500 V; nebulizer gas (N_2_), 2.8 bars; dry gas (N_2_), 8 Lmin^-1^; and dry heater, 200°C. Internal calibration was performed on the high-precision calibration mode (HPC) with a solution of sodium formate 10 mM introduced to the ion source *via* a 20-µl loop, at the beginning of each analysis using a six-port valve. Acquisition was performed in full scan mode in the *m/z* 70–1,000 range and in a data-depending MS/MS mode, with an acquisition of 5 Hz using a fixed cycle time of 3 s, and a dynamic exclusion duration of 0.4 min. Data acquisition and processing were performed by Data Analysis 4.2 software (Bruker Daltonics).

## 3 Results

Before real hydrolysates were used as substrates for the PHA production, the best operational conditions that maximize growth and accumulation of P3HB by *Halomonas boliviensis* in fed-batch cultivations were carried out with glucose.

### 3.1 Fed-batch assays with glucose to determine the best strategy to improve Poly-3-hydroxybutyrate productivity using phosphorous or nitrogen limitation

Two different strategies to promote polymer production were tested, namely, nitrogen limitation (N-lim) and limitation by phosphorous (P-lim). The P-limiting strategy has been successfully used by our group to trigger the P3HB accumulation in *Burkholderia sacchari* cultivations and was examined here (Cesário et al., 2014). The initial medium composition was similar to the one used by García-Torreiro et al. (2016) except for the NH_4_Cl and K_2_HPO_4_ concentrations. These were calculated based on mass balance equations and the average elemental bacterial cell composition (CH_1.92_O_0.3_N_0.24_P_0.02_) (Yeung, 2010) to attain a final biomass concentration of 20 g/L. This concentration was chosen here to demonstrate the proof-of-concept, that is, to show that P3HB can be produced from industrial residues of *Gelidium corneum* by *H. boliviensis* using either an N- or P-limiting approach to trigger polymer production.

The cultivation was performed in two stages. In the first stage, conditions were created to promote cell growth to attain a biomass concentration of circa 20 g/L, while the second stage aimed at polymer formation upon the exhaustion of one nutrient (N or P). Oxygen titers of 20% sat were maintained throughout the cultivation.

**FIGURE 1:**
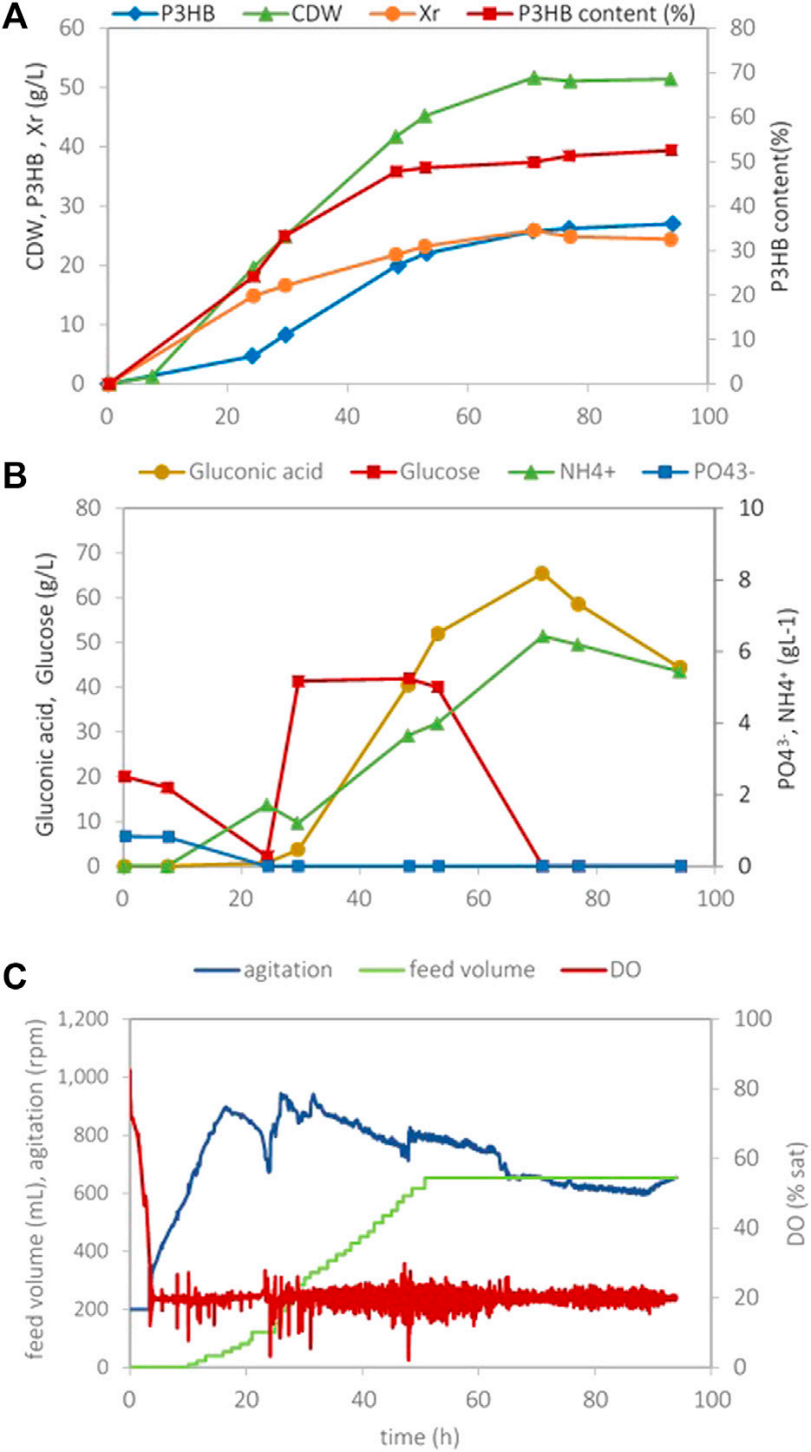
Data obtained in *H. boliviensis* fed-batch cultivation with mineral medium supplemented with 25 g/L of glucose, 1.5 g/L K_2_HPO_4_, and 7.0 g/L NH_4_Cl at pH 7.5 and 20% of DO. A solution containing 600 g/L of glucose (0.5 L) was used as feed. **(A)** Cell dry weight, P3HB concentration, the residual cell concentration (Xr), and the P3HB cell content during the cultivation, **(B)** glucose, gluconic acid, phosphate, and ammonia concentrations, and **(C)** data acquired automatically during the cultivation.

Figure 1 depicts the evolution of cultivation parameters using a P-lim strategy. The assays started with a K_2_HPO_4_ concentration of 1.5 g/L, which enabled the formation of a residual biomass concentration (*Xr*) of 20 g/L. The residual biomass concentration was calculated by the difference between the total biomass dry weight and the concentration of polymer (*Xr* = CDW- P3HB). An ammonium chloride concentration of 7 g/L was added to avoid double nutrient limitation.

Cell growth took place mainly until exhaustion of the phosphorous before 24 h cultivation; thereafter, a slight biomass increase was still observed until 53 h cultivation to a value of circa 24 g/L. This may probably be ascribed to the use of an intracellular phosphorous storage. The P3HB accumulation increased to approx. 26 g/L and halted upon glucose exhaustion. The P3HB content attained in this cultivation was 53%. The maximum overall volumetric productivity calculated from the time of inoculation (*Prod*_*vol,max*_) was 0.41 g_P3HB_ (L.h), and it was reached at 53 h. If only the period after exhaustion of the P source is considered, i.e., the production period, the maximum volumetric productivity was 0.83 g_P3HB_ (L.h). The overall yield of P3HB on total consumed glucose for growth and production was *Y*_P/S_ = 0.1 g_P3HB_/g_glucose_. P-lim assays were carried out in triplicate, and the average values attained are given in Table 2.

**TABLE 2:** Summary of the results obtained for *H. boliviensis* fed-batch cultivations in a bioreactor using P-lim and N-lim strategies toward P3HB production. Maximum P3HB titers attained, corresponding residual biomass (*Xr*), maximum overall volumetric P3HB productivity (*Prod*_*vol,max*_ (g/(L.h)), maximum specific P3HB productivity (*Prod*_*sp*_ (g_P3HB_/g_cell_ h)), yield of P3HB on glucose (*Y*_P/S_ (g_P3HB_./g_glucose_)) and yield of gluconic acid on glucose (*Y*_gluc acid/S_ (g_gluc acid_/g_glucose_)). Both yields were calculated for the same time period.

Type of limitation	*CDW*_*max*_ (g/L)	P3HB_max_ (g/L)	% P3HB (g_P3HB_/g_CDW_)	*Xr* (g/L)	*Prod*_*vol,max*_ [g/(L.h)]	*Prod*_*sp*_ [g_P3HB_/(g_cell_.h)]	*Y*_P/S_ (g_P3HB_/g_glucose_)	*Y*_gluc acid/S_ (g_gluc acid_/g_glucose_)
N-lim	22.8 ± 1.5	11.7 ± 0.9	51.1 ± 1.3	11.2 ± 0.6	0.12 ± 0.02	0.011 ± 0.02	0.15 ± 0.02	0.72 ± 0.03
P-lim	50.5 ± 5.9	27.2 ± 3.6	53.5 ± 0.9	23.6 ± 2.8	0.42 ± 0.03	0.020 ± 0.03	0.11 ± 0.02	0.25 ± 0.07

**FIGURE 2:**
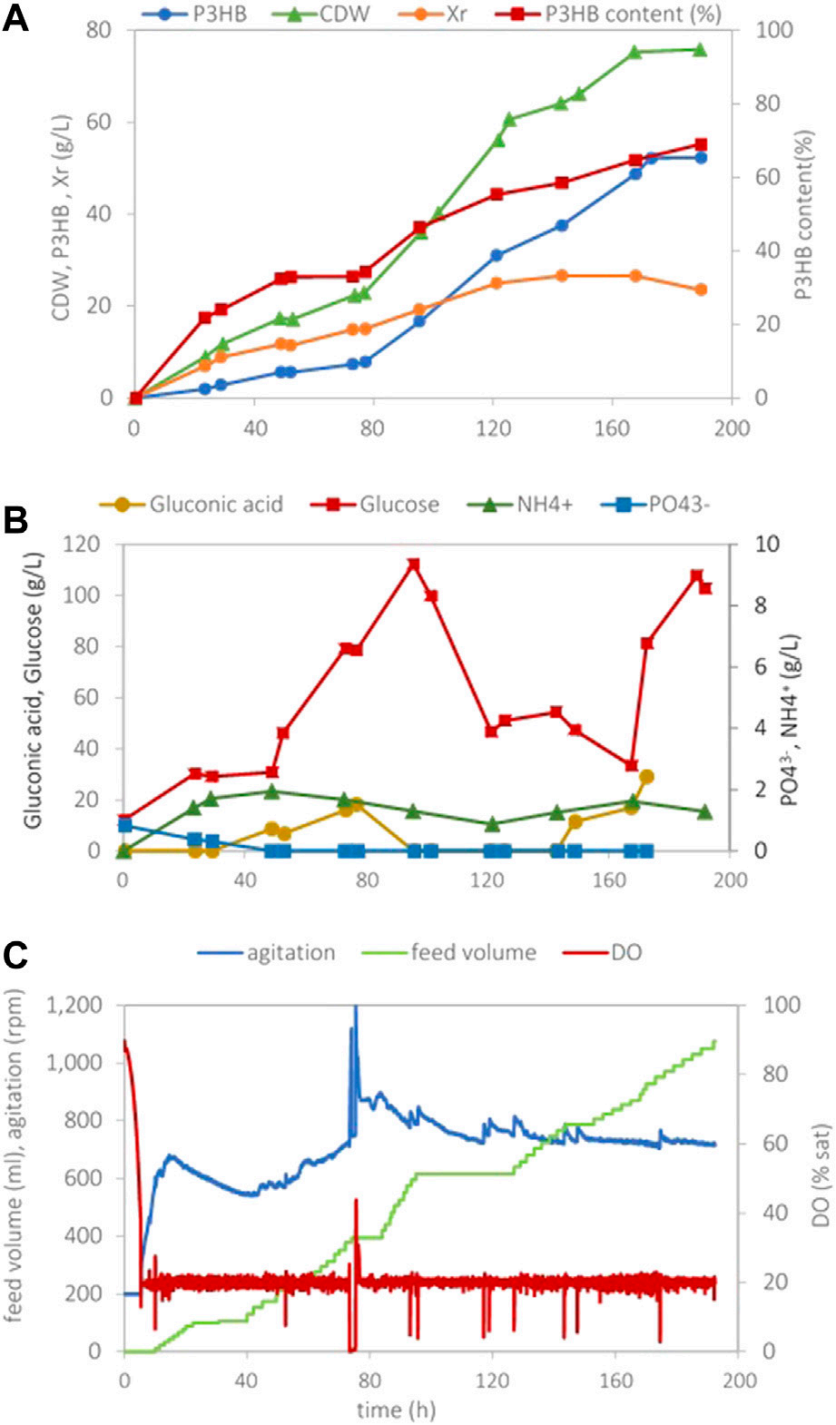
Data obtained in *H. boliviensis* fed-batch cultivation with mineral medium supplemented with 25 g/L of glucose, 1.5 g/L K_2_HPO_4,_ and 7.0 g/L NH_4_Cl at pH 7.5 and 20% sat of DO. A solution containing 600 g/L of glucose (1L) was used as feed. **(A)** Cell dry weight, P3HB, the residual cell concentration, and the P3HB cell content during the cultivation, **(B)** glucose, gluconic acid, phosphate, and ammonia concentrations, and **(C)** data acquired automatically during the cultivation.

The low yield value (*Y*_P/S_) attained in these assays is probably due to early exhaustion of the substrate, with most glucose being consumed for cell growth. To assess this, a cultivation was carried out (Figure 2) using the same initial nutrient concentrations but feeding a larger volume (1 L) of a 600 g/L glucose solution to reach a higher accumulation. In those conditions, a residual cell concentration of 24 g/L, similar to cultivation depicted in Figure 1 (as expected), was attained, and a maximum polymer concentration of 52 g/L and polymer content of 69% were obtained. This cultivation suffered a delay in cell growth after 50 h cultivation (not explainable) with consequently lower values of the overall maximum volumetric productivity [0.3 g/(L.h)] and the volumetric productivity during the production time [0.54 g/(L.h)]. The overall yield of polymer on glucose was 0.17 g _P3HB_/g _glucose_ as anticipated.

**FIGURE 3:**
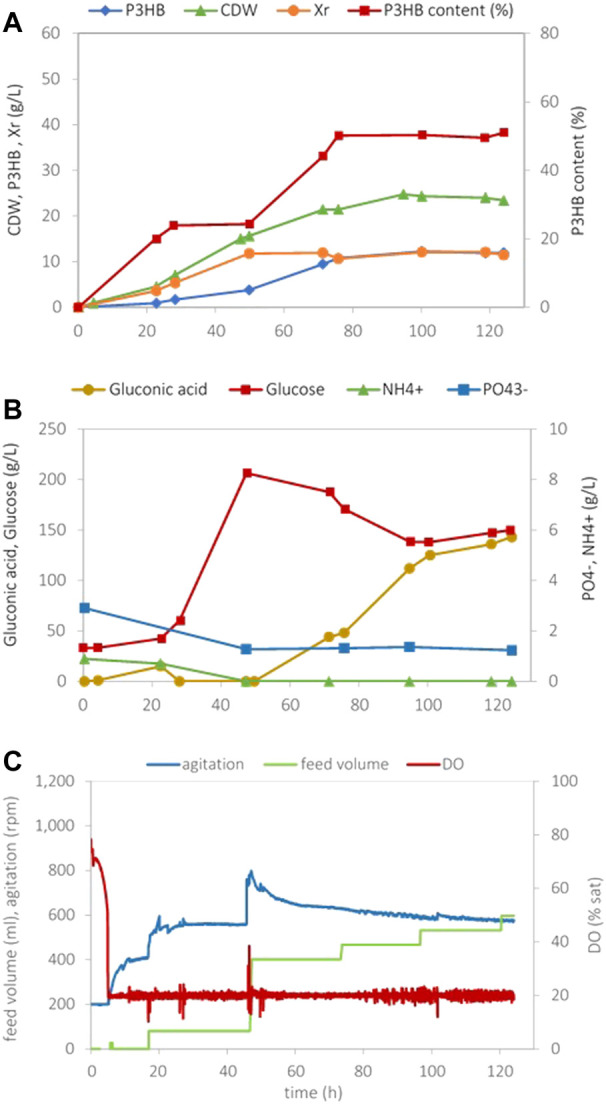
Data obtained in *H. boliviensis* fed-batch cultivation with mineral medium supplemented with 25 g/L of glucose, 4.5 g/L K_2_HPO_4_, and 3.0 g/L NH_4_Cl at pH 7.5 and 20% sat of DO. A solution containing 600 g/L of glucose was used as feed. **(A)** Cell dry weight, P3HB, the residual cell concentration, and the P3HB cell content during the cultivation, **(B)** glucose, gluconic acid, phosphate, and ammonia concentrations, and **(C)** data acquired automatically during the cultivation.

To compare these results with assays featuring N-limitation (N-lim), fed-batch cultivations with initial NH_4_Cl and K_2_HPO_4_ concentrations of 3 g/L and 4.5 g/L, respectively, were carried out. The results are given in Figure 3.

The cell growth took place until exhaustion of the NH_4_^+^ nutrient to a maximum *Xr* value of 11.8 g/L. Upon exhaustion of the N source (around 30 h cultivation), P3HB production was promoted, and a concentration of approx. 12 g/L was achieved, ceasing upon glucose exhaustion. The P3HB content attained in this cultivation was approx. 50%. The maximum overall volumetric productivity calculated considering the whole cultivation period (*Prod*_*vol,max*_) was 0.1 g_P3HB_/(L.h). If only the period after exhaustion of the N source was considered, i.e., the production period, the maximum volumetric productivity was 0.23 g_P3HB_/(L.h). The overall yield of P3HB on total consumed glucose was *Y*_P/S_ = 0.13 g_P3HB_/g_glucose_, which is similar to the values attained under P-limitation. N-lim assays were carried out in triplicate, and the average values attained are given in Table 2.

### 3.2 Identification of organic acids produced by *Halomonas boliviensis* during growth on glucose

Production of some organic acids was observed while growing *H. boliviensis* on glucose. These compounds were detected when monitoring the HPLC glucose consumption during cultivation.

**FIGURE 4:**
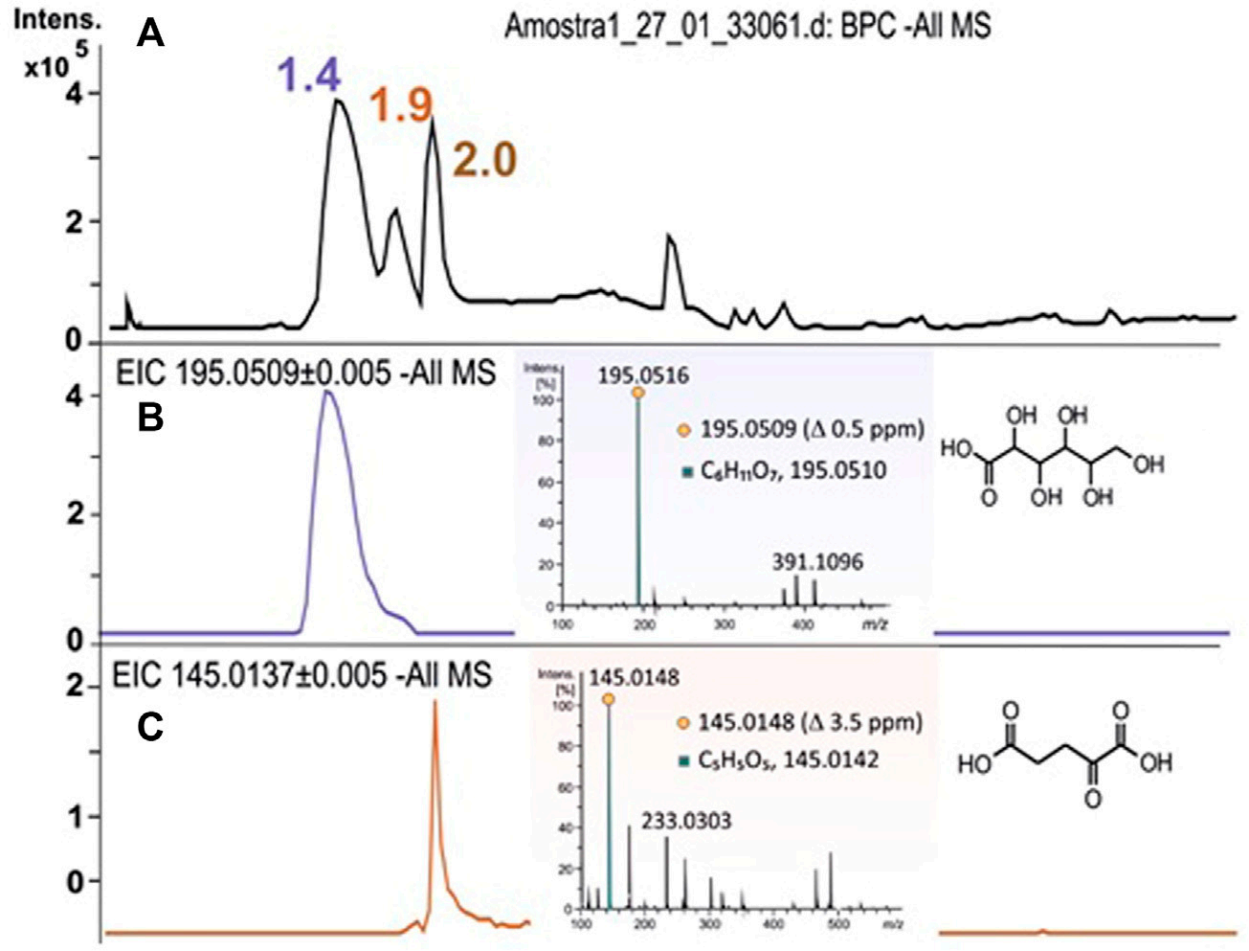
LC-HRMS analysis of an aliquot of the culture medium of the cultivation of *H. boliviensis* on glucose: **(A)** Total ion chromatogram obtained in the ESI-negative mode; the extracted ion chromatogram and HRMS spectrum for the deprotonated molecule of **(B)** gluconic acid: t_*R*_ 1.4 min, *m/z* 195.0509 (∆ −0.5 ppm, mSigma 5.5) (C_6_H_11_O_7_)^−^ and **(C)** 2-oxoglutaric acid: t_*R*_ 1.9 min, *m/z* 145.0148 (∆ −3.5 ppm, mSigma 8.5) (C_5_H_5_O_5_)^−^.

High-resolution mass spectrometry methodologies were applied to identify these compounds in several aliquots of the culture media along the cultivations. No significant signals were detected in the ESI-positive mode. Representative mass spectra in the ESI negative mode are shown in Figure 4. Two main signals were detected with m/z 195.0509 and 145.0148 assigned to the gluconic and 2-oxoglutaric acids, respectively. Furthermore, each precursor ion was isolated by using the quadrupole mass analyzer, transferred to the collision cell where CID experiments were performed, and the fragments were separated by using the TOF mass analyzer, leading to the MS/MS spectra. The peak assignment was based on the accurate m/z values released as deprotonated molecules (M-H)^−^, and each molecular formula was validated by extracting the ion chromatogram from the raw data. The accurate mass, isotopic pattern, and fragmentation paths were evaluated taking into account the accuracy and precision of the measurement parameters, such as error and sigma, and confirmed by comparison with standards or the literature data. The assignment of the fragment ions and the fragmentation paths are proposed in Supplementary Figure S1 presented as supplementary information.

### 3.3 Effect of the oxygen concentration on the production of gluconic acid and the yield of polymer in sugar

As gluconic acid is synthesized concomitantly with P3HB, its production from glucose by *H. boliviensis* most probably reduces the polymer yield. Because gluconic acid is a direct product of glucose oxidation, the influence of the oxygen concentration on the gluconic acid production and the P3HB yield was assessed. Different assays featuring phosphorous limitation and different concentrations of oxygen in the medium, namely, 5%, 20%, and 45% sat, were tested. The results are given in Table 3. It is clear that a higher oxygen concentration in the cultivation medium promotes the gluconic acid production and also decreases the yield of polymer on glucose. In view of these results and aiming at higher polymer productivities, further cultivations with *H. boliviensis* were carried out with phosphorous limitation and low oxygen concentrations.

**TABLE 3:** Summary of the results obtained for *H. boliviensis* fed-batch cultivations in a bioreactor using P-lim conditions and different oxygen concentrations.

DO-set point (% sat)	*CDW*_*max*_ (g/L)	*P3HB*_*max*_ (g/L)	*% P3HB* (g_P3HB_/g_CDW_)	*Xr* (g/L)	*Prod*_*vol*_ _max_ [g/(L.h)]	*Gluconic acid* (g/L)	*Y*_P/S_ (g_P3HB_/g_glucose_)
5	49.5.0 ± 4.9	30.4 ± 0.0.7	62.2 ± 7.3	18.9 ± 5.3	0.45 ± 0.03	30.2 ± 27.1	0.20 ± 0.02
20	50.5 ± 5.9	27.2 ± 3.6	53.5 ± 0.9	23.6 ± 2.8	0.42 ± 0.03	65.7 ± 0.42	0.11 ± 0.02
45	47.8	18.1	37.8	29.7	0.23	72.0	0.11

### 3.4 Poly-3-hydroxybutyrate production by *Halomonas boliviensis* based on residual *Gelidium* hydrolysates

Sugar-rich hydrolysates prepared from industrial residues of *Gelidium* biomass after agar extraction were successfully used by our study group as carbon sources for the growth and PHA production by *H. boliviensis* in shake flask assays (Tuma et al., 2020). These hydrolysates were here assayed in bench-scale (2L) bioreactors aiming at higher productivities. The conditions (determined in the paragraphs mentioned previously) that maximize polymer yields and productivities were applied, namely, phosphorous-limiting conditions and a DO of 5% saturation. Figure 6 shows the evolution of the cultivation. A maximum P3HB concentration and cell contents of 21.5 g/L and 41%, respectively, were attained. The polymer production halted at 47 h cultivation time due to exhaustion of glucose in the cultivation medium (glucose_feed_ = 305 g/L). A maximum overall polymer volumetric productivity of 0.46 g (L.h) was attained. As expected, a lower gluconic acid production during the cultivation was observed due to the low dissolved oxygen set-point used. As a control, an assay using commercial glucose simulating hydrolysate sugar concentrations both in the batch phase and feed was carried out, and the results are also given in Figure 6. Similar bacterial growth and production patterns can be observed when comparing both carbon sources, indicating that the hydrolysates from *Gelidium* residues can efficiently replace commercial glucose.

## 4 Discussion

*Halomonas boliviensis* DSM 15516, a moderately halophilic, alkali-tolerant, and psychrophilic bacterium, was isolated from the soil around a Bolivian hypersaline lake by Quillaguamán et al. (2004). These authors have carried out numerous bioreactor cultivations both in batch and in fed-batch mode and tested different sources of C and N. Organic N sources like yeast extract and monosodium glutamate (MSG) or inorganic sources such as ammonium chloride (NH_4_Cl) have been tested. To determine the best composition of a defined mineral medium, fed-batch cultivations in a bioreactor using glucose and different initial concentrations of NH_4_Cl and K_2_HPO_4_ were reported (Quillaguamán et al., 2008), and their effect on P3HB and the cell concentration were assessed. Monosodium glutamate was added because it was found necessary to support initial *H. boliviensis* growth (Balderrama-Subieta and Quillaguamán, 2013). In some assays, P3HB contents of 57% were observed, while in others maximum attained polymer contents of approximately 90% were reported. In the latter cases, the high polymer accumulations were mainly caused by the decrease of the residual cell concentration, as can be derived from the given data. In those assays, polymer production was promoted by exhaustion of nitrogen in the medium (N-limiting conditions). Maximum volumetric polymer productivities of 1.1 g/L.h were attained and were calculated based on the whole cultivation time.

García-Torreiro et al. (2016) have also carried out bioreactor cultivations with *H. boliviensis* aiming at high P3HB productivities. These authors have tested N- and oxygen-limitation strategies but have attained better results in cultivations featuring N-limitation and low oxygen supply (dissolved oxygen = 20% sat). Maximum productivities of 1.2 g/L.h were calculated considering only the polymer production period.

In the present study, the cultivations under N-limiting conditions (Figure 3) have been performed with an initial NH_4_Cl concentration of 3 g/L, aiming to attain a final residual biomass concentration (*Xr*) of 20 g/L. This concentration was calculated based on the typical elemental bacterial composition. However, only 12 g/L biomass was formed, and consequently, a lower polymer concentration and volumetric productivity were attained. A higher NH_4_Cl concentration should, thus, have been used to attain 20 g/L biomass, suggesting that the N stoichiometric coefficient used in the calculations does not apply to *H. boliviensis*. To conclude whether it is more efficient, in terms of P3HB production, to impose N- or P-limitation, the maximum specific productivity [*Prod_sp_*; (g_P3HB_/g_cell_.h)] was calculated as this parameter is independent of the cell concentration. These values are presented in Table 2. It is possible to observe that by limiting the P source in the medium, the specific polymer productivity approximately doubles as compared to the nitrogen-limiting condition. Therefore, P-limitation appears to be a more efficient strategy when using *H. boliviensis*.

During the cultivation of *H. boliviensis* on glucose, the production of some organic acids was observed. Although 2-oxoglutaric acid was identified in culture samples, its concentration was negligible, in contrast with gluconic acid that was produced in high titers. In fact, gluconic acid concentrations as high as 143 g/L (Figure 3) were attained. To the best of the authors’ knowledge, the ability of *Halomonas boliviensis* to synthesize gluconic acid is here reported for the first time.

**FIGURE 5:**
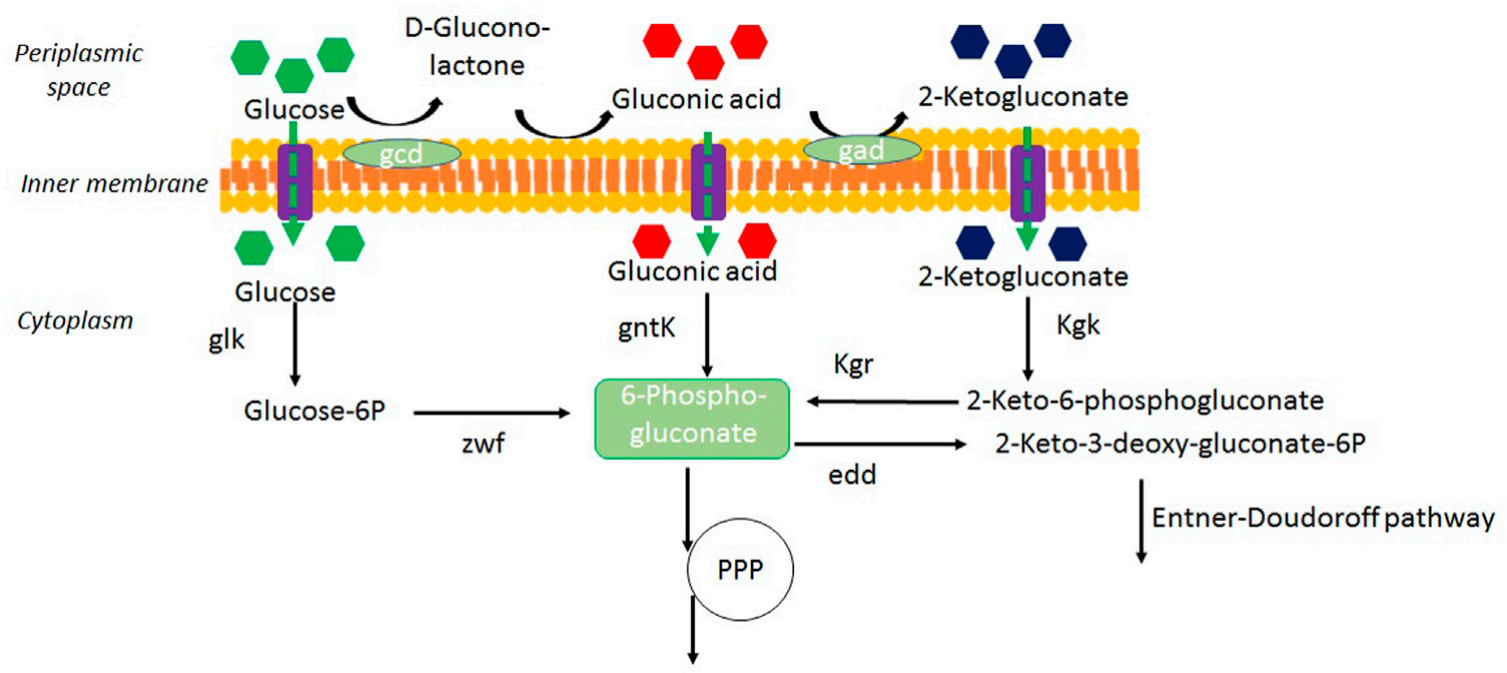
Alternative pathways of gluconic acid formation from glucose in *H. boliviensis* DSM 15516. Glucose utilization can follow the direct oxidative pathway in the periplasmic space to gluconic acid or enter the cytoplasm and follow the phosphorylative pathway to produce glucose-6-P. The abbreviations stand for PPP, the pentose-phosphate pathway; gcd, glucose dehydrogenase; gad, gluconate dehydrogenase; gntK, gluconokinase; kgk, 2-ketogluconokinase; kgr, 2-keto-6-phosphogluconate reductase; zwf, glucose-6P dehydrogenase; glk, glucokinase; edd, glucose-6P dehydratase.

The presence of gluconate in the culture medium indicates the capacity of *H. boliviensis* to catabolize glucose using the gluconate pathway, which involves the oxidation of glucose to gluconate through D-glucono-1,5-lactone (Figure 5) and takes place in the periplasmic space. When glucose is exhausted in the medium, *H. boliviensis* is able to consume this acid, as observed in Figures 1, 2. This fact indicates that gluconate might be directly consumed by the PP metabolic pathway or through the formation of 2-ketogluconate, which is further metabolized by the Entner–Doudoroff (E-D) pathway (Figure 5). According to Rivera-Terceros et al. (2015), the genome sequence of *H. boliviensis* revealed the existence of genes coding for enzymes involved in the E-D and PP pathways. However, no genes were found related to the expression of 6-phosphogluconate dehydrogenase which catalyzes the conversion of 6-phospho-d-gluconate into ribulose-5-phosphate through the PP pathway (Figure 5). For this reason, 6-phosphogluconate is probably transformed into 2-keto-3-deoxy-6P-gluconate and subsequently cleaved into pyruvate and glyceraldehyde-3-phosphate by the E-D pathway (Ramachandran et al., 2006).

The presence of gluconate in the cultivation medium suggests a possible overflow of metabolism. This could arise from limited catabolism of glucose and thus a reduced demand, leading to higher excretion of gluconate (Pastor et al., 2013).

The metabolite 2-oxoglutarate (also known as α-ketoglutarate, 2-ketoglutaric acid, or oxoglutaric acid) is an intermediate of the Krebs cycle and is the point of intersection between the carbon and nitrogen metabolism. The increase of the oxoglutarate concentration in the cultivation medium was observed to be associated with pulse additions of glutamate (data not shown). This metabolite is produced by the deamination of glutamate by the enzyme glutamate-dehydrogenase, constituting an anaplerotic reaction that replenishes the TCA cycle at this stage: glutamate + NAD^+^ + H_2_O → NH_4_^+^ + α-ketoglutarate + NADH + H^+^.

Glutamate metabolism in *H. boliviensis* was discerned based on genome sequence analysis (Balderrama-Subieta and Quillaguamán, 2013).

The yield of gluconic acid on glucose is given in Table 2. *Y_gluc acid/S_* (g_gluc acid_/g_glucose_) was calculated for the same time period as the yield of P3HB (*Y*_P/S_; g_P3HB_/g_glucose_). For both cultivation strategies, the yield of gluconic acid on glucose is higher than that of P3HB and this is even more clear when using the N-limitation strategy for P3HB production. Although the production of gluconic acid is not the focus of this study, because glucose is being diverted for the production of this acid instead of polymer, the effect of oxygen concentration was here assessed. The results regarding the effect of the oxygen concentration in the medium indicated that higher oxygen concentrations promote the gluconic acid production, while a decrease in the polymer concentration, polymer accumulation (% P3HB), and thus the yield of polymer on sugar (*Y*_P3HB/glucose_) was observed. These results corroborate those by Quillaguamán et al. (2005) where major enhancements in the P3HB yield were reported when oxygen limitation was induced in a fermenter.

It is believed that the production of gluconic acid by *H. boliviensis* has not been reported in previous studies because most authors have used a glucose kit to follow glucose consumption. Because gluconic acid is a commercially valuable product and this study showed that *H. bolivienis* is a good producer, cultivation conditions that facilitate gluconic acid production will be assessed in the future.

**FIGURE 6:**
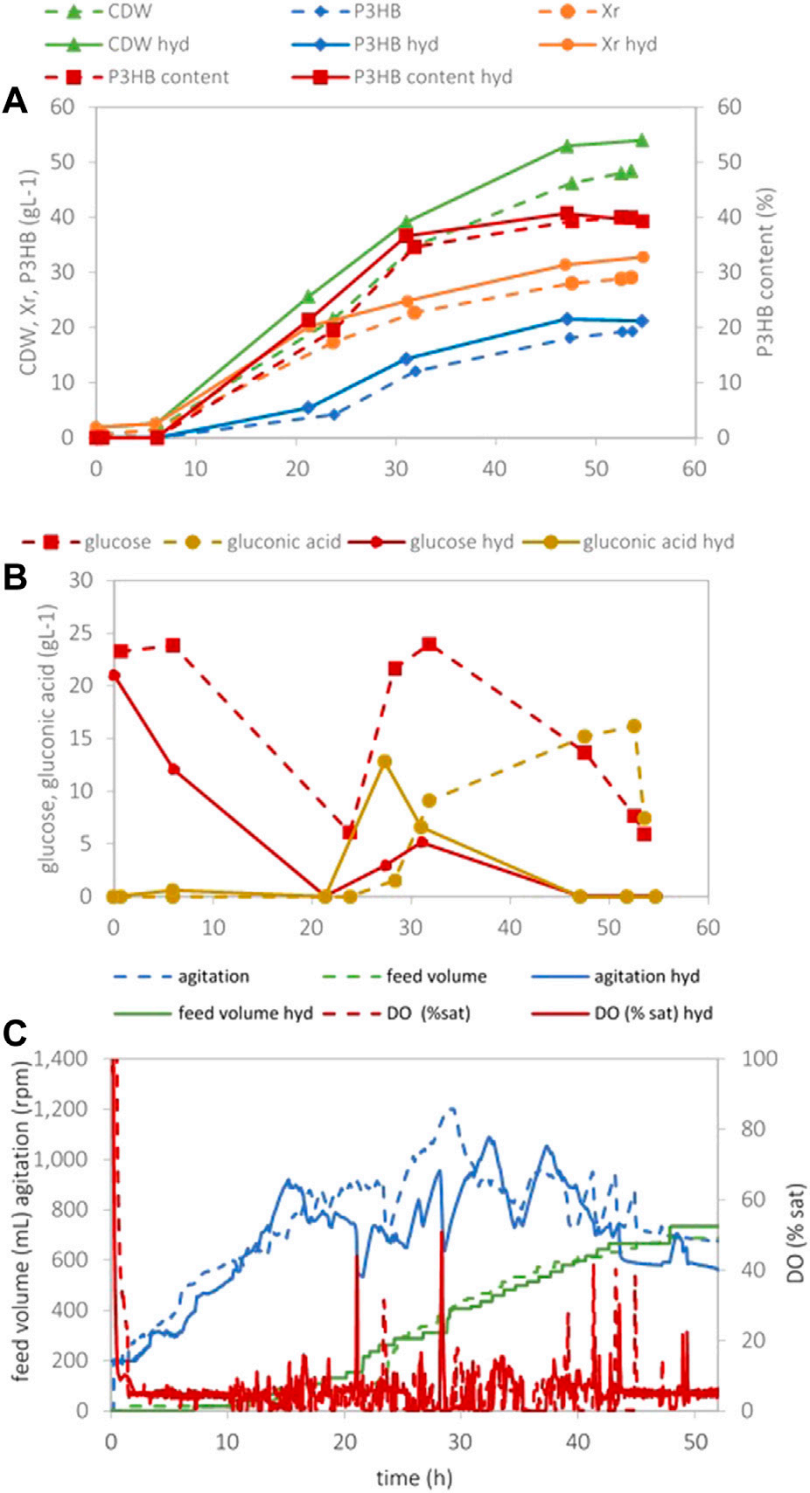
Data obtained in *H. boliviensis* fed-batch cultivation using glucose or hydrolysates of *Gelidium* residues in the batch and the feed–mineral medium supplemented with 25 g/L of glucose (dashed line) or hydrolysate (solid line), 1.5 g/L K_2_HPO_4_, and 7.0 g/L NH_4_Cl at pH 7.5 and 5% sat of DO. A 500 ml solution containing 305 g/L of glucose and 45 g/L NaCl or the hydrolysate supplemented with 45 g/L NaCl was used as feed. **(A)** Cell dry weight, P3HB, residual cell concentration, and P3HB cell content during the cultivation, **(B)** glucose and gluconic acid concentrations, and **(C)** data acquired automatically during the cultivation.

When using real *Gelidium* hydrolysates, a maximum P3HB concentration, cell content, and maximum overall volumetric polymer productivity of 21.5 g/L, 41%, and 0.46 g/(L.h), respectively, were attained, while the simulated fermentation with glucose achieved values of 19.3 g/L, 40%, and 0.38 g/(L.h), respectively (Figure 6). These results are very similar and slightly higher in the case of the algal hydrolysate, clearly demonstrating the feasibility of using residual *Gelidium* biomass as a sugar platform for the production of polyhydroxyalkanoates. Data on fed-batch cultivations using algal hydrolysates as the carbon source for the production of PHAs are scarce in the literature. Alkotaini et al. (2016) have assessed the use of *Gelidium amansi* hydrolysates prepared by acid hydrolysis as C-source in fed-batch cultivations with *Bacillus megaterium*. When the concentrated hydrolysate was fed using the intermittent feeding strategy, a PHA concentration, content, and productivity of 5.5 g/L, 54.5%, and 0.09 g/(L.h), respectively, were attained. These values are much lower than the values attained in the present study, except for the PHA content that is reasonably higher. In that study, the lower values are due to the lower sugar and higher inhibitor concentrations due to the simple acid treatment applied for producing the hydrolysates, without steps for enzymatic hydrolysis and inhibitor removal.

This study showed that limiting the concentration of phosphorous in the medium is a very effective strategy to promote poly-3-hydroxybutyrate accumulation by *Halomonas boliviensis* cultivations, leading to competitive polymer productivities and yields. It is worth noting that this approach is particularly relevant when using biomass hydrolysates as a carbon source for polymer production since these feedstock will always contain N-rich components such as peptides and proteins that promote bacterial growth instead of polymer production.

During the present study, it has been observed for the first time that *H. boliviensis* produces two organic acids, when growing on glucose. These acids have been identified as gluconic and oxo-glutaric acids. Gluconic acid production occurs simultaneously with polymer production, causing a decrease in the yield of polymer on glucose. Lowering the dissolved oxygen concentration in the cultivation medium caused a decrease in the gluconic acid production and improved P3HB titers and yields.

Industrial *Gelidium corneum* residues after agar extraction were shown to be good carbon platforms for polyhydroxyalkanoate production. After being processed to glucose-rich hydrolysates, this waste biomass was upgraded to P3HB by *H. boliviensis* with high yields and productivities, similar to the ones attained with commercial glucose. The proof-of-concept of using waste algal biomass for the production of poly-3-hydroxybutyrate with high yields, titers, and productivities was validated in this work.

## Data Availability

The original contributions presented in the study are included in the article/Supplementary Material; further inquiries can be directed to the corresponding author.
