# Supplementary material for: Red algae industrial residues as a sustainable carbon platform for the co-production of poly-3-hydroxybutyrate and gluconic acid by Halomonas boliviensis
Source: Front Bioeng Biotechnol. 2022 Oct 10;10:934432. doi: 10.3389/fbioe.2022.934432 (PMC9588912; doi:10.3389/fbioe.2022.934432)
Supplement: Supplementary file 1 [file DataSheet1.docx]

**Supplementary Figure 1:** Fragment ions and the fragmentation paths of gluconic acid (Scheme 1S) and 2-oxoglutaric acid (Scheme 2S).

Scheme 1S- Proposed fragmentation pattern for the deprotonated molecule of gluconic acid *m/z* 195.0509 [C_6_H_11_O_7_]^-^.

Scheme 2S- Proposed fragmentation pattern for the deprotonated molecule 2-oxoglutaric acid *m/z* 145.0148 [C_5_H_5_O_5_]^-^.
